# Enhanced Bandgap Flexibility in Perovskite‐Silicon Tandem Solar Cells via Three‐Terminal Architecture

**DOI:** 10.1002/advs.202520603

**Published:** 2026-01-29

**Authors:** Mohammad Gholipoor, Michael Rienaecker, Xuzheng Liu, Seyedamir Orooji, Lingyi Fang, Paul Fassl, Renjun Guo, Uli Lemmer, Robby Peibst, Ulrich Wilhelm Paetzold

**Affiliations:** ^1^ Light Technology Institute (LTI) Karlsruhe Institute of Technology (KIT) Karlsruhe Germany; ^2^ Institute of Microstructure Technology (IMT) Karlsruhe Institute of Technology (KIT) Eggenstein‐Leopoldshafen Germany; ^3^ Institute for Electronic Materials and Devices Leibniz University Hannover Hannover Germany; ^4^ Institute for Solar Energy Research Hamelin (ISFH) Emmerthal Germany

**Keywords:** perovskite, photovoltaics, POLO, tandem solar cells, three terminal

## Abstract

Monolithic perovskite/silicon tandem photovoltaics are among the most promising high‐efficiency technologies for next‐generation photovoltaics. However, the commercial development of two‐terminal (2T) tandem configurations is limited by their operational instability of wide‐bandgap perovskite materials, which leads to current mismatch and increased sensitivity to solar spectral variations. Three‐terminal (3T) tandem architectures offer a viable route to address these limitations. Here, we demonstrate the real‐world advantages of 3T perovskite/silicon tandem solar cells in mitigating current mismatch limitations and losses arising from solar spectral variations. Our 3T tandem solar cells achieve a power conversion efficiency of 30.1%, integrating a front‐side textured interdigitated back contact (IBC) and poly‐Si on oxide contact (POLO) silicon bottom cell. This is one of the highest efficiencies reported for 3T tandem solar cells so far. Through a direct comparison of 2T and 3T tandem configurations enabled by a novel measurement framework, we reveal that 3T architectures decouple performance from perovskite bandgap constraints, alleviating the need for the current matching. Additionally, 3T tandem solar cells exhibit enhanced spectral resilience under varying solar spectra when the top cell limits the short‐circuit current. These findings underscore the potential of 3T architectures for stable and efficient tandem photovoltaics under real‐world operating conditions.

## Graphical abstract

Three‐terminal (3T) perovskite/silicon tandem solar cells overcome the current‐matching constraints of 2T designs. Using an IBC POLO silicon bottom cell, we demonstrate a 30.1% PCE, with performance largely decoupled from perovskite bandgap and enhanced resilience under realistic, time‐dependent solar spectra.

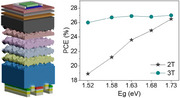

## 1 Introduction

Recent advances have elevated the power conversion efficiency (PCE) of perovskite/silicon tandem solar cells (PSTSCs) to 34.6% [1], surpassing the Shockley–Queisser limit for single‐junction solar cells. This milestone has generated significant optimism for PSTSCs as a transformative technology in photovoltaics [2, 3, 4]. However, these PCE records are primarily achieved through two‐terminal (2T) architecture‐based technology, requiring stringent current matching between the subcells [5, 6, 7, 8]. This limitation also exposes PSTSCs to efficiency losses under real‐world conditions [9, 10, 11], such as temporal and weather‐related spectral variations [12, 13, 14], as well as top PSCs degradation [4, 15]. Furthermore, the strict requirement for bandgap optimization in 2T designs restricts material flexibility, limiting the exploration of alternative bandgap perovskite compositions [16, 17, 18, 19]. Addressing these challenges is critical for advancing PSTSC's commercialization and meeting global renewable energy targets.

Although the four‐terminal (4T) PSTSCs can eliminate current‐matching constraints, they are hindered by system integration challenges, including balance‐of‐system (BOS) disadvantages [20], encapsulation complexity [3], a larger dead area due to laser scribing [21], and significant parasitic absorption losses occurring in the thick transparent conductive oxide (TCO) layer [22, 23]. Three‐terminal (3T) tandem architectures offer a promising alternative by combining the advantages of 2T and 4T designs while minimizing their drawbacks [24, 25, 26, 27, 28]. 3T tandem solar cells(TSCs) are typically fabricated in two configurations [29]: one incorporating a middle contact and the other employing interdigitated back contact (IBC) silicon solar cells [30]. However, the middle contact configuration requires either a thick TCO layer [31]. or a metal grid electrode [26], which increases parasitic absorption and fabrication complexity. In contrast, IBC silicon bottom cells eliminate the need for a middle contact, enabling the top cell to be monolithically integrated, similar to 2T PSTSCs. This streamlined approach reduces optical losses and simplifies the manufacturing process, making it a promising solution for high‐efficiency tandem solar cells. Additionally, the IBC configuration is one of the main technologies used for reporting PCEs over 27% and above in silicon photovoltaics (PV) [32, 33] and is currently gaining commercial market share [34]. Also, a well‐established 3T tandem interconnection, so‐called voltage‐matched string, was developed for 3T module integration [35, 36, 37]. The design, compromising a mixture of series and parallel connections, allows subcells to operate at their MPP with less sensitivity to spectral variation. Although this 3T string interconnection suffers from complexity, string end losses, and increased cabling costs [38], its practical application has been experimentally proven to be even superior to 2T tandem strings [39].

Here, we present a front‐side textured 3T PSTSC featuring poly‐Si on oxide (POLO) IBC silicon bottom cells, a configuration compatible with industrial standards. Poly‐Si‐based passivated contacts are considered the most promising and dominating (from 2024 on) silicon technology due to their >26% efficiency [40], scalability, and mass production adaptability [41, 42]. We achieve a PCE of 30.1%, comparable to the prior records of 29.11% [26] and 29.56% (certified in 2T mode) reported for 3T solar cells [39, 43], using the same iterative measurement technique as in those studies [29, 44, 45]. Consistent with previous simulation studies [44, 45], we experimentally confirm that the 3T architecture decouples TSCs' performance from perovskite bandgap constraints, removing the need for precise current matching. Moreover, we show that 3T solar cells are less sensitive to spectral variations than 2T TSCs under top‐cell‐limited conditions, resulting in higher power generation during weather fluctuations. In agreement with prior studies [25], our numerical study also demonstrates that 3T TSCs achieve a higher annual energy yield (EY) under various climatic conditions than the 2T architecture, confirming their greater robustness to spectral variations for long‐term application. Beyond this established understanding, we add that the higher EY of 3T TSCs compared to their 2T counterparts becomes more evident in sunnier locations than cloudier environments, particularly in greater current mismatches. These findings highlight the potential of 3T POLO‐based tandem architectures to address critical challenges in PSTSCs and accelerate their deployment in real‐world applications.

## 2 Results and Discussion

**FIGURE 1:**
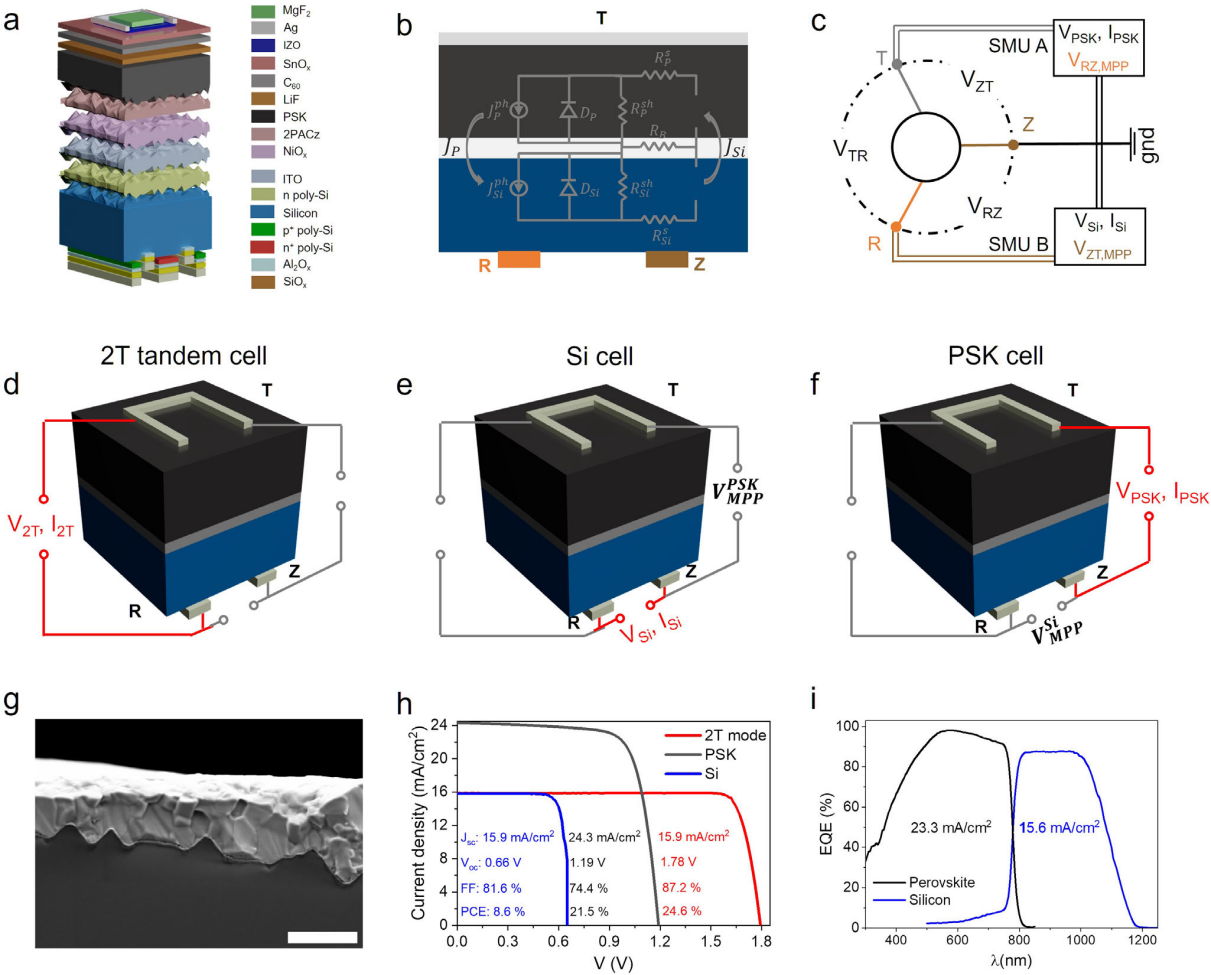
3T‐PSTSCs' measurement method and champion cell performance. (a) Device architecture of the 3T‐TSC, illustrating the double‐sided textured perovskite top cell integrated with a poly‐Si on oxide (POLO) interdigitated back contact (IBC) silicon bottom cell. (b) Corresponding equivalent electrical circuits of 2T and 3T structures. The RB resistor is assumed to be absent in 2T mode and present in 3T mode. (c) A representation of the cycle of iterative measurement. The terminologies for the contacts (T, Z, and R) were introduced by Warren et al., [29]. (d–f) Sketches of the 3T architecture, containing rear R and Z contacts and T front contact with all required external connections. Biasing T‐R connection allows the device to operate in a 2T mode, while biasing the Z–T and R–Z connections approximately enables independent measurement of the perovskite top and silicon bottom cells, respectively. (g) The SEM cross‐section image. a solution‐processed perovskite (metallic gray) covering on sub‐micro c‐Si textures (dark area). The scale bar is 2 µm. h, Current−voltage (J−V) characteristics of the top and bottom subcells of the champion 3T TSC, along with 2T TSC's results. i, The corresponding external quantum efficiency (EQE) spectra of the perovskite (black) and silicon (blue) subcells in the tandem device, and the integrated J_SC_ of the corresponding subcells.

To meet industrial scalability and mass production standards, 3T‐TSCs must incorporate monolithically integrated subcells, similar to 2T‐PSTSCs. Figure 1a illustrates the architecture of our 3T‐TSCs. The silicon bottom cell features POLO‐junctions for all three contacts. While there are various 3T tandem configurations [29], we chose a “PVK (Perovskite)/s/nuIBC” structure for the following reasons: at least one side of the bottom cell needs to be textured with random pyramids to ensure sufficient light trapping and, consequently, absorption in the long‐wavelength regime. Implementing the surface texture on the rear side, as common in most 2T architectures, is challenging in an IBC structure with n‐ and p‐type fingers on the rear. We therefore texture the front side while keeping the rear‐side planar. We use sub‐micrometre‐sized random pyramids to be compatible with wet chemical processing of the perovskite top cell [46]. Due to the superior passivation quality of n‐type doped POLO contacts on textured surfaces (as compared to their p‐type doped counterparts [47]), we apply an electron‐collecting nPOLO junction on the front. For a p‐i‐n perovskite top cell monolithically deposited on the bottom cell, this results in a series‐type (“s”) subcell interconnection. In our device, it is realized by an indium tin oxide (ITO) recombination layer. The n‐type wafer doping (“nIBC”) ensures a uni‐junction (“u”) type bottom cell with only contact collecting minority carriers (holes in our case). The alternative—a bipolar junction type bottom cell with two minority carrier collecting contacts based on a p‐type doped wafer—was found to suffer from injection‐level dependent minority carrier transport losses in previous studies [48].

To accurately evaluate the performance of 3Tsolar cells, it is essential to account for the interdependent behavior of the subcells. To achieve this, we apply two source measure units (SMUs) to precisely monitor the distribution of voltages and currents across the two active subcells [29, 37]. Figure 1c–f illustrates the electrical wiring diagram and subcells measurement strategy, respectively. The rear electrical contacts, Z and R, function as electron‐selective and hole‐selective terminals, respectively, while the front T contact serves as an electron‐selective contact, forming an n‐i‐p top cell configuration. When only T‐R connection is biased, the device operates in a 2T mode (Figure 1d). Also, when the Z–T and R–Z connections are biased, the perovskite top and silicon bottom cells can approximately be independently measured, respectively (Figure 1e,f). The third contact (Z) enables the extraction of surplus electrons into the Si bottom cell when the top cell produces less current than the Si bottom cell, or the injection of missing electrons from the Si bottom cell when it generates less current [29]. This type of three‐terminal interconnection topology is called a “common Z” configuration (V_ZT_, V_RZ_). The respective unused contact is grounded for these measurements.

To accurately measure the total 3T‐TSC's PCE, both subcells (ZT and RZ connections) must operate simultaneously at their maximum power point (MPP) voltages. This is essential because, in a 3T configuration, the subcells interact interdependently, such that biasing one subcell affects the performance of the other. Toward this purpose, the subcells are characterized iteratively. First, the maximum power point voltage (V_MPP_) of the perovskite top solar cell is determined while the silicon bottom solar cell is held at open‐circuit. Then, the TZ contact is biased at its measured V_MPP_ to determine the V_MPP_ of the RZ contact, as illustrated in Figure 1e. This process is repeated until both subcells' performance stabilizes, ensuring no further changes in bias voltages. This method allows for the precise determination of each subcell's MPP under illumination, enabling accurate calculation of the 3T device efficiency as the sum of the subcell efficiencies. This method accounts for the electrical and optical interplay of both subcells and is comparable in accuracy to a more complex sweeping of all possible V_ZT_ ‐V_RZ_ combinations [49].

From the perspective of the equivalent circuit [50], during current mismatch, the Z contact introduces a new electrical node and an associated resistance (R_B_) into the circuit (Figure 1b), effectively resolving the challenges associated with the monolithic series connection of the subcells. According to Kirchhoff's current law, the third branch allows the current imbalance between the subcells to either leave or enter the circuit, ensuring continuous and efficient operation.

Subsequently, according to the above‐mentioned iterative method, the subcells were measured to obtain 3T tandem solar cell performance. Also, the tandem device is measured in 2T mode for comparison. The champion 3T tandem cell achieves an impressive overall PCE of 30.1%, provided as the sum of the PCEs of the individual subcells, as shown in Figure 1h. The achieved PCE is as high as the highest efficiency reported to date for 3T tandem solar cells [39]. The perovskite top cell features a composition of Cs_0.05_MA_0.22_FA_0.73_Pb(I_0.90_Br_0.10_)_3_, with an optical bandgap (E_g_) of ∼1.58 eV. This bandgap was randomly selected for an attempt to achieve a champion efficiency of 3T tandem solar cells. The cross‐sectional scanning electron microscopy (SEM) image confirms that the micrometer‐thick perovskite layer uniformly covers the sub‐micrometer‐textured silicon bottom cell (Figure 1g). The Si bottom solar cell (RZ) of this champion device exhibited a PCE of 8.6%, with a open‐circuit voltage (V_OC_) of 0.66 V, a fill factor (FF) of 81.6%, and a short‐circuit current density (J_SC_) of 15.9 mA/cm^2^. Meanwhile, the perovskite top solar cell (TZ) achieved a PCE of 21.5%, with a V_OC_ of 1.19 V, an FF of 74.4%, and a J_SC_ of 24.3 mA/cm^2^. This high performance was achieved by minimizing parasitic absorption losses and effectively passivating the interface between the C_60_ and the perovskite film by 1,3‐propane‐diammonium iodide (PDAI_2_) (Figures S1 and S2). Table 1 summarizes the V_MPP_ results for each step, highlighting the iterative approach's effectiveness. Notably, the performance of the champion cell in the two‐terminal (2T) configuration lags significantly behind its 3T performance due to a substantial current mismatch between the subcells for the specific perovskite bandgap (E_g_ = 1.58 eV). Figure 1i shows the corresponding external quantum efficiency (EQE) spectrum, which corroborates the J_SC_ values obtained from the J–V measurements, further validating the results. The slight difference between the J_SC_ values derived from EQE and J–V measurements arises from a technical offset in the low‐ and high‐wavelength regions of the AM 1.5 spectrum, slightly overestimating J_SC_ of perovskite subcells in 3T and the FF in 2T solar cells.

**TABLE 1:** Device performance and iterative MPP.

	Bias Condition other subcell (V)	PCE (%)	FF (%)	V_oc_(V)	J_SC_ (mA/cm^2^)	V_MPP_ (V)
Perovskite	OC	20.9	73.8	1.17	24.3	0.93
	0.55	21.0	73.9	1.17	24.3	0.94
	0.56	21.5	74.4	1.19	24.3	0.95
Silicon	OC	8.7	81.8	0.63	16.9	0.55
	0.94	8.6	81.4	0.66	15.9	0.56
	0.95	8.6	81.6	0.66	15.9	0.55
2T (TR measurement)	FW	24.6	86.9	1.78	15.9	
	BW	24.6	87.2	1.78	15.9	
	2T (average FW/BW)	24.6				
	3T measurement	30.1				

The most significant advantage of the 3T configuration is to eliminate the strict requirement for optimal bandgaps in the constituent subcells. By excluding the necessity of a defined bandgap for subcells, we are able to utilize perovskite bandgaps with greater stability and efficiency. For instance, this allows the use of stable α‐phase perovskites [51] instead of mixed‐halide perovskites, which suffer from halide phase segregation [52]. In 2T‐TSCs, the ideal current‐matching condition is primarily determined by the bandgap (E_g_) of the perovskite top cell, which typically lies in the range of 1.65–1.70 eV. However, this optimal bandgap is subject to variations due to factors such as operational temperature fluctuations and parasitic optical losses inherent in layered device structures. Consequently, 2T‐TSCs are constrained to specific perovskite bandgaps, limiting material flexibility and hindering the exploration of alternative compositions. For instance, while Br‐rich perovskites with E_g_ values of 1.65–1.70 eV achieve current matching, they often exhibit poor stability, whereas perovskites with an E_g_ of ≈1.5 eV, such as FAPbI_3_, have shown significantly improved stability in recent years. These constraints motivate our investigation into 3T architectures, which can circumvent these limitations.

**FIGURE 2:**
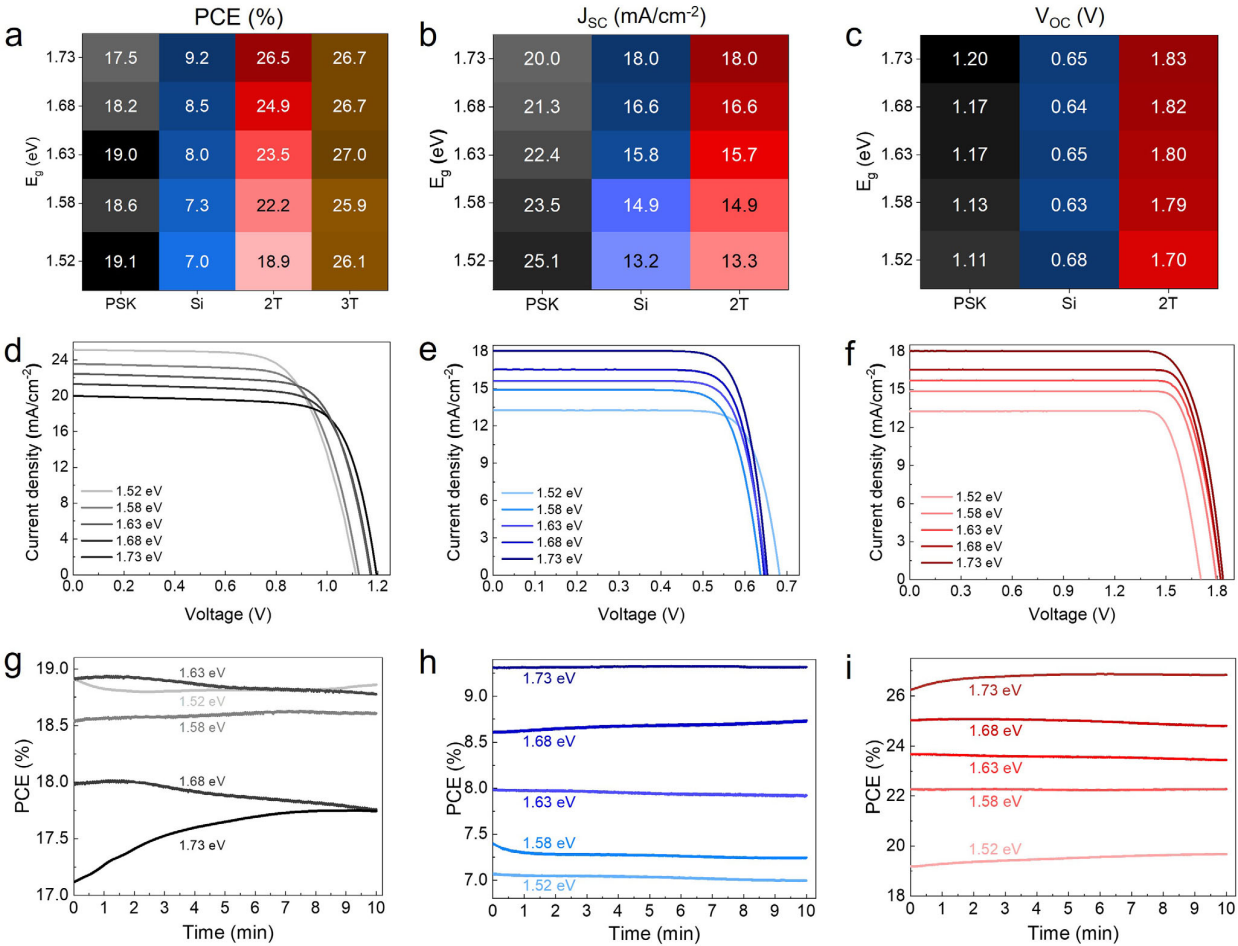
The measured performance of 2T and 3T‐PSTSCs for five different perovskite bandgaps of the top cell (1.58–1.73 eV). (a–c) Solar cell parameters (PCE, J_SC_, and V_OC_) of each subcell in the 2T and 3T tandem solar cells. The lighter and darker color expresses the lower and higher respective performance parameters. (d–f) Current−voltage (J−V) curves of the Si and perovskite subcells as well as the corresponding 2T‐TSC for five different perovskite bandgaps. Here, the brightness of each color changes with the bandgap of perovskite, such that a darker color is indicative of a wider bandgap and vice versa. (g–i) The 10‐min MPP track of non‐encapsulated subcells and 2T‐TSCs in air. Temperature and humidity were not controlled, 20–30°C and 25–35%, respectively. Similarly, the color of each bandgap becomes darker with the increase of the perovskite bandgap.

To address this, we fabricated tandem solar cells with five different top‐cell bandgaps (1.52–1.73 eV). Unlike Figure 1, where the passivation layer was PDAI_2_, for these five perovskite top subcells, we utilized LiF as the passivation layer for the subsequent stability tests, since LiF showed better stability than PDAI_2_ in our study (Figure S3). Figure 2a–c illustrates three performance parameters (PCE, J_SC_, and V_OC_) of the silicon bottom solar cell, perovskite top solar cell, and the corresponding 2T‐TSCs for five different bandgaps (perovskite composition and fabrication see Supporting Information). Figures S4 and S5 demonstrate the SEM images and XRD patterns of all five perovskite surfaces. The FF of corresponding cells is shown in Figure S6. The changes in the relative brightness of each color are indicative of a cell performance alteration. Figure 2d–f show the respective J‐V curves of the same cells. The corresponding EQE spectrums of each bandgap agree with the J_SC_ derived from the J–V scans (Figure S7). The results show that the performance of the Si bottom solar cell improves as the perovskite bandgap increases, primarily due to an increase in theJ_SC_. A wider perovskite bandgap allows more light to pass through and be absorbed by the Si layer, enhancing its performance. Conversely, the performance of the perovskite top solar cell decreases with increasing bandgap, mainly due to reduced light absorption and J_SC_. Additionally, wide‐bandgap perovskites exhibit higher V_OC_ deficits, which limit the V_OC_ gain and do not fully compensate for the J_SC_ reduction. As a result, the overall PCE of the 2T TSCs increases with the perovskite bandgap. For absolute values, we remark that both subcells—as well as the interface in between—can be improved. For the latter, we observe that the sputtering of the ITO recombination layer compromises the passivation quality of the poly‐Si on oxide on the front bottom cells, causing a V_OC_ loss of > 50 mV. This issue can be addressed in future works by applying soft sputtering processes or TCO‐free subcell interconnection schemes. In contrast, the efficiency of 3T‐TSCs is independent of the perovskite bandgap, as is reflected by the negligible brightness changes of the 3T column (brown color) in Figure 2a and Figure S8. The 3T configuration allows the subcells to operate without the need for current matching, enabling the use of a broader range of perovskite bandgaps. This versatility implies that the design of the 3T architecture is much more flexible, allowing one to select the optimal perovskite bandgap free of current matching constraints. The latter is particularly relevant to stability and durability. For example, more stable perovskite compositions, such as ≈1.5 eV FAPbI_3_, can be employed without compromising performance. Moreover, the efficiency of 3T‐TSCs is calculated as the sum of the PCEs of the two subcells, providing a straightforward method for performance evaluation. These results show the ability of the 3T configuration to overcome the bandgap limitations of 2T‐TSCs, enabling greater flexibility and improved stability in PSTSCs.

Figure 2g–i exhibits the 10‐min stability of non‐encapsulated all five Si bottom cells, perovskite top solar cells, and 2T solar cells in ambient. The separate stability track of both subcells provides clearer details about the contribution of each subcell to the tandem cell degradation. The perovskite top cells are relatively unstable under illumination compared to the silicon bottom cells. Also, the stability of perovskite is aggravated with the bandgap increase, which can arise from the increase of the Br/I ratio. However, in a tandem solar cell, the instability of perovskite is compensated to some extent by silicon bottom cells. Additionally, we tracked the stability of the current density of all bandgaps (Figure S9). To further investigate the response of 2T and 3T TSCs to changes in the perovskite bandgap, we present the box charts of photovoltaic performance metrics (PCE, FF, V_OC_, and J_SC_) for a limited number of 2T solar cells with varying bandgaps in Figures S10–S12. The corresponding J–V curves of individual subcells and 2T TSCs are provided in Figures S13–S17. Also, the iterative measurement of subcells presents further details, such as V_MPP_ changes and subcell interplay, about each bandgap subcell and tandem cell performance for different bandgaps (Tables in Figures S13–S17). In 2T solar cells, the PCE increases with the perovskite bandgap due to enhancements in both J_SC_ and V_OC_. The V_OC_ improvement reflects a more significant quasi‐Fermi level splitting (QFLS) for wider bandgaps, while the J_SC_ increase is attributed to improved current matching between the subcells. However, the influence of J_SC_ on 2T‐TSCs' PCE is more pronounced than that of V_OC_, as discussed previously. As the TSC deviates further from the ideal bandgap matching point (E_g_ = 1.73 eV), the detrimental impact of J_SC_ reduction in the limiting subcell becomes dominant, significantly impairing 2T solar cell performance. In cases of severe current mismatch, a slight compensation is observed through an increase in FF, but this is insufficient to offset the reductions in J_SC_ and V_OC_, rendering 2T solar cells‐TSCs impractical for non‐optimized bandgaps. In contrast, 3T solar cells are unaffected by subcell current mismatch, as all photogenerated carriers from both the perovskite and Si layers are collected independently. The total J_SC_ extracted from a 3T solar cell is the summation of the J_SC_ values from both subcells, eliminating current‐mismatch losses. The results underscore the flexibility and superior performance of 3T devices across a wide range of perovskite bandgaps compared to their 2T counterparts.

**FIGURE 3:**
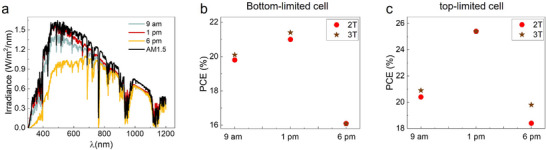
2T and 3T PSTSCs under varying clear sky irradiance spectra. (a) Spectral irradiance of a solar simulator emulating three different sunlight spectra corresponding to specific times of the day at a given location. The spectra illustrate variations in light intensity and distribution, particularly in the visible region. (b) Corresponding performance of 2T and 3T solar cells under the spectral conditions shown in (a) and (c) when the tandem cell is bottom‐limited. (c) Corresponding performance of 2T and 3T solar cells under the same spectral conditions when the tandem cell is top‐limited. Additional IR LEDs were used to adjust the spectral composition, converting the operating condition of the tandem device from bottom‐limited to top‐limited.

The performance of 2T and 3T TSCs can differently vary under varying sunlight spectra, particularly when considering the operational conditions of top‐limited and bottom‐limited tandem configurations. To demonstrate the difference between the robustness of 2T and 3T‐TSCs against varying spectra, we analyzed the performance of 2T and 3T solar cells under sunlight spectra at three distinct times of day—morning, noon, and evening— in Phoenix (arid/desert location) of the USA for a specific date. The irradiance data of Phoenix are obtained from the typical meteorological year (TMY3) [53], and then corresponding spectra are computed by a basic cloud model and SmartCode [54, 55] (Figure S18). Figure 3 highlights how these spectral changes impact device performance, revealing that the response of 2T and 3T configurations to sunlight variations depends on whether the tandem cell is top‐limited or bottom‐limited. This occurs because high‐energy photons (shorter wavelengths) are more susceptible to absorption or scattering under varying weather conditions, leading to reduced carrier generation in the top solar cell. This reduction limits the current output of 2T‐TSCs, as their performance is constrained by the current of the limiting subcell. In contrast, 3T tandem solar cells, which operate independently of current matching, exhibit greater resilience to spectral variations and distribute the photogenerated carriers more effectively between the subcells. When a tandem cell is top‐limited, as shown in Figure 3c, 2T solar cells demonstrate a stronger dependence on sunlight spectral changes compared to their 3T counterparts. This highlights the ability of 3T solar cells to generate more power consistently throughout the day, making them more robust for long‐term performance. It is worth noting that top‐limited conditions are more likely due to the pronounced variability in shorter‐wavelength sunlight during the day. Additionally, perovskite‐specific issues, such as light‐induced degradation and phase segregation, can exacerbate top‐limited conditions by further widening the perovskite bandgap. In such scenarios, 3Tsolar cells are better equipped to maintain stable performance over time, providing a significant advantage for solar tandem farms. These findings underscore the long‐term viability of 3T solar cells over 2T configurations in real‐world operating environments. When a tandem cell is bottom‐limited, a reduction in lower‐wavelength light intensities does not significantly impact the total current of the 2T solar cells. This is because the transition from a bottom‐limited to a top‐limited state does not result in a substantial change in the subcell current difference. As a result, the performance differences between 2T and 3T solar cells are minimal in bottom‐limited tandem cells, as shown in Figure 3b. To investigate both top‐limited and bottom‐limited scenarios using the same tandem cell and to validate this hypothesis, we selected a slightly bottom‐limited solar cell. Under conditions of reduced sunlight intensities, this configuration enabled us to study the bottom‐limited case as described above. Additionally, to simulate the top‐limited case, we introduced an extra infrared (IR) LED to the setup, shifting the same cell to a slightly top‐limited state. This approach allowed us to examine and compare the device performance in both limiting conditions within a controlled environment.

**FIGURE 4:**
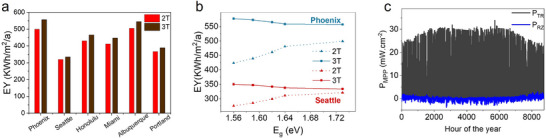
EY performance of 2T and 3T‐TSCs in different climatic conditions. (a) The simulated annual energy yield of 2T and 3T solar cells in different locations. The perovskite bandgap was selected at 1.73 eV to meet the best experimental current matching. (b) Bandgap‐dependent annual energy yield of 2T and 3T solar cells in the sunniest location (Phoenix) and the cloudiest location (Seattle). These results highlight the superior spectral resilience of 3T solar cells, which is especially pronounced in sunnier locations. Unlike 2T solar cells, which experience significant efficiency losses due to current mismatch between subcells, 3T solar cells maintain stable performance across varying spectral conditions. (c) Power generation profile in the RZ and RT circuits of the 3T solar cell in Phoenix for a perovskite layer with a band gap of 1.73 eV.

The diurnal and seasonal variations in solar spectra under outdoor field‐testing conditions significantly impact the annual EY of solar cells. These variations, driven by differences in location and weather, play a critical role in determining the long‐term performance of tandem devices. Due to their dependence on current matching, 2T solar cells are more sensitive to these spectral fluctuations compared to 3T solar cells, which are designed to operate independently of subcell current limitations. We calculate the annual EY of 2T and 3T solar cells across various locations representing diverse climatic zones in the United States. Figure 4a shows the simulated EY for seven locations, using a perovskite bandgap of 1.73 eV to optimize current matching in 2T‐TSCs, as validated experimentally (Figure S19 and Table S1). Across all locations, 3T architectures consistently outperform 2T devices in annual EY. However, the performance gap between the two configurations varied by location. In areas with more direct sunlight, such as Phoenix, the advantage of 3T devices is more pronounced, with an EY of 546 kWh/m^2^/a for 3T cells compared to 513 kWh/m^2^/a for 2T cells. Conversely, in regions with predominantly diffuse light, such as Seattle, the difference is minimal, with EYs of 329 and 327 kWh/m^2^/a for 3T and 2T devices, respectively. This variability can be attributed to the diurnal and spectral fluctuations in light intensity. Locations with greater variations in light intensity amplify the performance differences between 2T and 3T devices, as 2T cells suffer from current mismatch losses under non‐optimal spectral conditions. More interestingly, when a bandgap of perovskite deviates from its ideal bandgap matched point, the difference in annual EY of 2T and 3T solar cells becomes more pronounced in sunnier locations than cloudier ones. Figure 4b shows the dependence of annual EY on perovskite bandgaps for 2T and 3T solar cells in Phoenix (the sunniest location) and Seattle (the cloudiest location). While 3T solar cells in Phoenix generate approximately 9 kWh/m^2^/a more annual EY than their 2T counterparts at a bandgap of 1.73 eV, this difference value increases dramatically to 89.5 kWh/m^2^/a for a smaller bandgap of 1.56 eV. On the other hand, in Seattle, as a cloudier region, this advantage grows more modestly–from 2 to 40 kWh/m^2^/a–when the perovskite bandgap changes from 1.73 to 1.56 eV. Thus, these results confirm that 3T solar cells deliver higher annual energy yields than 2T devices when the perovskite bandgap shifts away from the ideal match, and this performance edge becomes markedly more dominant in regions with abundant sunlight. Consequently, the lower annual EY of 2T devices across all locations is primarily driven by energy losses due to the current mismatch, showing the robustness and efficiency of 3T architectures in diverse environmental conditions, more specifically in sunnier regions. further investigate how 3T‐TSCs can generate higher annual power under realistic outdoor conditions, we calculate the hourly power generation profile over an entire year under two electrical circuits‐TR and RZ. The results for Phoenix are presented in Figure 4c, with data for other locations provided in Figure S20. While the TR circuit is the primary source of annual electrical power generation, the RZ circuit actively enhances overall power output throughout the year by compensating for current mismatch throughout the year. P_RZ_ contributes to overall power generation when a current mismatch occurs at the MPP points of subcells. When J_MPP,Si_ > J_MPP,Pe_, the RZ circuit generates power (P_RZ_ > 0) by extracting excess photogenerated carriers. Conversely, when J_MPP,Pe_ > J_MPP,Si_, the Z contact injects electrons in the bottom cell, leading the RZ circuit to supply power (P_RZ_<0). However, as previously mentioned in the introduction, any losses arising from supplying P_RZ_ are fully compensated by an increased power generation in the TR circuit. Thus, in both scenarios, the RZ circuit plays a crucial role in enhancing the total energy output of 3T devices, demonstrating their ability to mitigate current mismatch losses and improve long‐term energy yield under realistic operational conditions.

## 3 Conclusions

In this study, we conduct a comprehensive analysis highlighting the superior performance and extended operational boundary of three‐terminal tandem solar cells compared to two‐terminal solar cells. For the first time, a front‐side textured interdigitated back contact POLO silicon cell representative for the current mainstream technology in Si photovoltaics, is integrated into a tandem architecture and measured in both 2T and 3T modes. Our results demonstrate that the current matching constraint inherent to 2T solar cells, which limits their adaptability and material choices, is eliminated in 3T architectures. Such flexibility allows 3T solar cells to exploit a broader range of perovskite bandgaps, enabling the integration of recent advancements in perovskite materials. To explore this potential, we systematically study the performance of tandem solar cells with perovskite bandgaps ranging from 1.52 to 1.73 eV. As expected, the performance of 2T solar cells is significantly constrained by the limiting perovskite subcell, whereas 3T solar cells show consistent performance across all bandgaps, demonstrating their independence from current matching. This capability ensures that 3T solar cells can accommodate diverse perovskite compositions, offering opportunities for improved stability and efficiency. Furthermore, we investigate the impact of varying sunlight spectra on device performance, particularly under top‐limited and bottom‐limited conditions. Our findings reveal that 3T tandem solar cells are substantially less sensitive to spectral changes when the tandem cell is top‐limited. Such a scenario is more likely due to the variability of shorter‐wavelength light throughout the day and the inherent stability challenges of perovskite materials. While the advantages of 3T solar cells are less pronounced under bottom‐limited conditions, the dominance of top‐limited scenarios during daily and seasonal sunlight variations suggests that 3T tandem solar cells can generate more electricity over the long term. These findings highlight the superior adaptability and robustness of 3T architectures for real‐world applications, where sunlight conditions fluctuate throughout the day and year.

## 4 Experimental Section/Methods

### 4.1 Ink Formulation for Perovskite Thin Film Deposition

Dimethyl sulfoxide anhydrous ≥99.9% (DMSO, CAS: 67‐68‐5), N,N‐dimethylformamide ≥99.9% (DMF, CAS: 68‐12‐2), and Ethyl Acetate anhydrous 99.8% (EA, CAS: 141‐78‐6) were purchased from Sigma Aldrich. Formamidinium iodide (FAI, CAS: 879643‐71‐7) purchased from Dyenamo. Methylamonium Bromide (MABr, CAS: 6876‐37‐5) was bought from Greatcell Solar. Lead iodide (PbI_2_, CAS: 10101‐63‐0) and Lead Bromide (PbBr_2_, CAS: 10031‐22‐8) were purchased from TCI. Cesium Iodide (CsI, CAS: 7789‐17‐5) was bought from abcr. Fullerene (C_60_, CAS: 99685‐96‐8) was purchased from Sigma‐Aldrich. 2PACz (CAS: 20999‐38‐6), was bought from TCI. Ethanol absolute 99.8% for the SAMs was bought from VWR Chemicals.

### 4.2 Silicon Subcell Preparation

The following fabrication process yields the 3T POLO^2^‐IBC bottom cell: A 2.2 nm‐thin interfacial oxide layer is thermally grown onto a single‐side nano‐textured 280 µm‐thick 3 Ω cm n‐type FZ wafer. The nano‐texture with pyramid heights below 1 µm was fabricated at SINGULUS TECHNOLOGIES AG by using an alkaline KOH solution with a surface activating additive [7]. We subsequently cap the interfacial oxide by a low‐pressure chemical vapor deposited intrinsic amorphous Si layer. We perform masked ion implantation of B and P into the amorphous Si on the rear side and a blanket P implantation on the front side. For the former, we pattern a SiO_x_ mask as an implant barrier by photolithography to result in interdigitated n^+^‐type and p^+^‐type fingers. Then, the amorphous Si recrystallizes during a wet oxidation to grow a thick SiO_2_ layer, and the POLO junctions form in a subsequent higher temperature process at 1035°C. We optimized the planar nPOLO and pPOLO junctions to excellent recombination pre‐factors of below 0.1 and 1.8 fA/cm^2^, respectively. The recombination pre‐factors of the nPOLO junction on the textured front side were minimized to 2.2 fA/cm^2^ after hydrogenation.

After annealing, we remove the p^+^n^+^ poly‐Si junction formed on the rear side by KOH etching a trench between the poly‐Si fingers. The thick SiO_2_ protects the front‐side nPOLO contact and the rear‐side pPOLO and pPOLO contacts during the KOH etching. Then the front‐side SiO_x_ is removed via single‐sided HF treatment, and the poly‐Si thickness on the front side is reduced to ∼35 nm using an isotropic etch in an ammonium‐peroxide mixture.

After removing the SiO_2_ from the rear side, we deposit a triple‐layer stack of AlO_x_/SiN_y_/SiO_z_ on the rear side to passivate the trench region, to hydrogenate the POLO junctions, and to facilitate the laser contact opening process. The front‐side receives an AlO_x_ layer for hydrogenation. After laser opening the contacts, we perform an HF dip to remove the AlO_x_ from the front side and potentially remaining AlO_x_ from the contact openings.

We sputter deposit an ITO bilayer, which provides a common interface for PVK cell processing. The ITO layer was sputtered using a 90/10 In_2_O_3_/SnO_2_ target (90 wt.% indium oxide, 10 wt.% tin oxide), with a purity of 99.99%. To minimize parasitic absorption in the front‐side ITO layer, we use a bilayered ITO structure, consisting of a 5 nm seed ITO layer sputtered without O_2_ flow, resulting in a high carrier density to ensure proper contact formation with the poly‐Si, followed by a 15 nm transparent ITO top layer reactively sputtered with O_2_.

After the ITO deposition, SiO_x_ isolation strips are sputtered onto the front side, and the bottom cell precursors are annealed at 300°C in a nitrogen atmosphere. Finally, we perform a rear‐sided HF treatment and evaporate a 10 µm‐thick Al layer and a SiO_z_ layer on the rear side, and perform the contact separation. The wafers are laser‐scribed on the rear side and cleaved into 25 × 25 mm^2^ substrates, yielding ∼1.1 cm^2^ bottom solar cells.

### 4.3 Perovskite Top Solar Cell Fabrication

Isopropyl alcohol and acetone were used to wash silicon substrates using a spin‐coater with (3000 rpm, 1000 rpm/s, 30 s). The top half‐stack is started with the deposition of 15 nm NiO_x_ by RF sputtering onto the planar ITO/Silicon substrates at room temperature with a base pressure of <3×10^−7^ Torr, RF power of 100 W, argon flow rate of 18 sccm for 10 min. Then, 0.5 mg/ml 2PACz solution in ethanol was deposited by spin‐coating in a nitrogen glovebox at 3000 rpm for 30 s and then annealed at 100°C for 10 min. To fully fill the sub‐micrometer‐sized pyramids, 1.7 M perovskite precursor solution was prepared with molecular formula:

$$\left(Cs_{0.15}FA_{0.85}\mathrm{Pb}I_{3}\right)\left(1.52\;\mathrm{eV}\right)$$

$$(Cs_{0.05}MA_{0.10}FA_{0.85}\mathrm{Pb}{\left(I_{0.90}Br_{0.10}\right)}_{3}\left(1.58\;\mathrm{eV}\right)$$

$$(Cs_{0.05}MA_{0.16}FA_{0.78}\mathrm{Pb}{\left(I_{0.83}Br_{0.17}\right)}_{3}\left(1.63\;\mathrm{eV}\right)$$

$$({\mathrm{Cs}}_{0.05}{\mathrm{MA}}_{0.22}{\mathrm{FA}}_{0.73}\mathrm{Pb}{\left(I_{0.77}{\mathrm{Br}}_{0.23}\right)}_{3}\left(1.68\;\mathrm{eV}\right)$$

$$({\mathrm{Cs}}_{0.05}{\mathrm{MA}}_{0.27}{\mathrm{FA}}_{0.67}\mathrm{Pb}{\left(I_{0.71}{\mathrm{Br}}_{0.29}\right)}_{3}\left(1.73\;\mathrm{eV}\right)$$

The perovskite layers were deposited by spin coating in a nitrogen glovebox at 400 rpm (400 rpm/s) for 3 s and 2000 rpm (2000 rpm/s^−1^) for 60 s, then 350 uL ethyl acetate as antisolvent was dropped 5 s before the end of the second step. Afterwards, the perovskite layers were annealed at 100°C for 30 min. After depositing the perovskite layer, approximately 1 nm of LiF was deposited via thermal evaporation. Then, 20 nm C_60_ was deposited by thermal evaporation. Subsequently, 20 nm SnO_2_ was deposited by atomic layer deposition. Utilizing radio‐frequency magnetron sputtering, a 90 nm IZO layer using a show mask was deposited on top of SnO_2_ layer. Then, 600 nm silver contacts in a C‐shape, along with fingers, were thermally deposited. Ultimately, 120 nm MgF_2_ anti‐reflection layer was thermally evaporated.

Simulation method: An in‐house developed energy yield modeling platform, EYCalc, is used to compute the EY of perovskite–silicon tandem solar cells. The software is published as an open‐source software project. It comprises four modules.

1. irradiance module: it computes the hourly direct and diffuse solar irradiance throughout the year at various locations across the USA using data from the typical meteorological Year. The module inputs meteorological data from the TMY3 dataset into a simplified atmospheric radiative transfer model to compute clear‐sky irradiance. Additionally, a basic cloud model is applied to account for weather variations.
2. optics module: The optics module uses the transfer‐matrix method to analyze optically coherent thin layers and applies a series expansion based on the Beer–Lambert law for optically incoherent thick layers. The module also can also simulate stacks with textured interfaces using geometrical ray‐tracing, as described by Baker–Finch and McIntos.
3. electrical module: The electrical module calculates the temperature‐dependent current density–voltage characteristics of perovskite–silicon tandem solar cells. Numerical simulations are conducted using a coupled two‐diode model in LTspice.
4. EY module: The core EY module calculates the energy yield of perovskite–silicon tandem solar cells over their entire lifetime, accounting for the module's orientation (rotation and/or tilt) and location. Temperature effects are inherently included through the nominal operating cell temperature model.

## Conflicts of Interest

The authors declare no conflicts of interest.

## Supporting information




**Supporting File**: advs73930‐sup‐0001‐SuppMat.docx.

## Data Availability

The data that support the findings of this study are available from the corresponding author upon reasonable request.
