# Supplementary material for: Enhanced Bandgap Flexibility in Perovskite‐Silicon Tandem Solar Cells via Three‐Terminal Architecture
Source: Adv Sci (Weinh). 2026 Jan 29;13(18):e20603. doi: 10.1002/advs.202520603 (PMC13042970; doi:10.1002/advs.202520603)
Supplement: Supplementary file 1 — Supporting File: advs73930‐sup‐0001‐SuppMat.docx. [file ADVS-13-e20603-s001.docx]

**Supplementary Material**

**Enhanced Bandgap Flexibility in Perovskite-Silicon Tandem Solar Cells via Three-Terminal Architecture**

Mohammad Gholipoor, Michael Rienaecker, Xuzheng Liu, Seyedamir Orooji, Lingly Fang, Paul Fassl, Renjun Guo*, Uli Lemmer, Robby Peibst*, and Ulrich Wilhelm Paetzold*

M. Gholipoor, X. Liu, S. Orooji, P. Fassl, R.Guo, U. Lemmer, U. W. Paetzold

Light Technology Institute (LTI), Karlsruhe Institute of Technology (KIT), Engesserstrasse 13, 76131, Karlsruhe, Germany
E-mail: [renjun.guo@kit.edu](mailto:renjun.guo@kit.edu) [peibst@isfh.de](mailto:peibst@isfh.de) [ulrich.paetzold@kit.edu](mailto:ulrich.paetzold@kit.edu)

M. Gholipoor, X. Liu, S. Orooji, L. Fang P. Fassl, R.Guo, U. Lemmer, U. W. Paetzold

Institute of Microstructure Technology (IMT), Karlsruhe Institute of Technology (KIT), Hermann-von-Helmholtz-Platz 1, 76344, Eggenstein-Leopoldshafen, Germany

M. Rienaecker, R. Peibst

Institute for Electronic Materials and Devices, Leibniz University Hannover, Schneiderberg 32, 30167, Hannover, Germany

R. Peibst

Institute for Solar Energy Research Hamelin (ISFH), Am Ohrberg 1, 31860, Emmerthal, Germany

Contents

[Supplementary Fig. 1│ Box comparison of performance of two samples before and after optimization. 4](#_Toc217914358)

[Supplementary Fig. 2│ comparison of J-V results of samples before and after optimization. 5](#_Toc217914359)

[Supplementary Fig. 3│ MPP Tracking of 2T tandem solar cell with LiF passivation (black) and PDAI_2_ passivation (red) 6](#_Toc217914360)

[Supplementary Fig. 4│ Top-view SEM images of perovskite films. 7](#_Toc217914361)

[Supplementary Fig. 5│ XRD patterns of the perovskite films. 8](#_Toc217914362)

[Supplementary Fig. 6│ FF changes of subcells, as well as 2T and 3T tandem cells, with five different perovskite bandgaps. 9](#_Toc217914363)

[Supplementary Fig. 7│ EQE spectra of the perovskite (black) and silicon (blue) subcells for different bandgaps. 10](#_Toc217914364)

[Supplementary Fig. 8│ Dependence of 2T and 3T performances on perovskite bandgaps. 11](#_Toc217914365)

[Supplementary Fig. 10│ Box comparison of one or two of perovskite subcell performance with different bandgaps. 13](#_Toc217914366)

[Supplementary Fig. 11│ Box comparison of one or two of silicon subcell performance for different bandgaps. 14](#_Toc217914367)

[Supplementary Fig. 12│ Box comparison of one or two of 2T tandem device performance for different bandgaps. 15](#_Toc217914368)

[Supplementary Fig. 13│ subcells, 2T, 3T devices performance data of 1.52 eV bandgap, along with their corresponding J-V data. 16](#_Toc217914369)

[Supplementary Fig. 14│ subcells, 2T, 3T devices performance data of 1.58 eV bandgap, along with their corresponding J-V data. 17](#_Toc217914370)

[Supplementary Fig. 15│ subcells, 2T, 3T devices performance data of 1.63 eV bandgap, along with their corresponding J-V data. 18](#_Toc217914371)

[Supplementary Fig. 16│ subcells, 2T, 3T devices performance data of 1.68 eV bandgap, along with their corresponding J-V data. 19](#_Toc217914372)

[Supplementary Fig. 17│ subcells, 2T, 3T devices performance data of 1.73 eV bandgap, along with their corresponding J-V data. Tables represent V_MPP_ changes and performance parameters in an iterative measurement. 20](#_Toc217914373)

[Supplementary Fig. 18│ solar simulator spectra 21](#_Toc217914374)

[Supplementary Fig. 19│ Measured EQE spectra (dotted line) and simulated EQE spectra (solid line) of subcells. 22](#_Toc217914375)

[Supplementary Table. 1│ Performance parameters of Measured and simulated EQE. 23](#_Toc217914376)

[Supplementary Fig. 20│ Power generation proﬁle in the RZ and RT circuits of the 3T solar cell in Seattle, Honolulu, Miami, and Albuquerque for a perovskite layer with a band gap of 1.73 eV. 24](#_Toc217914377)

1 µm


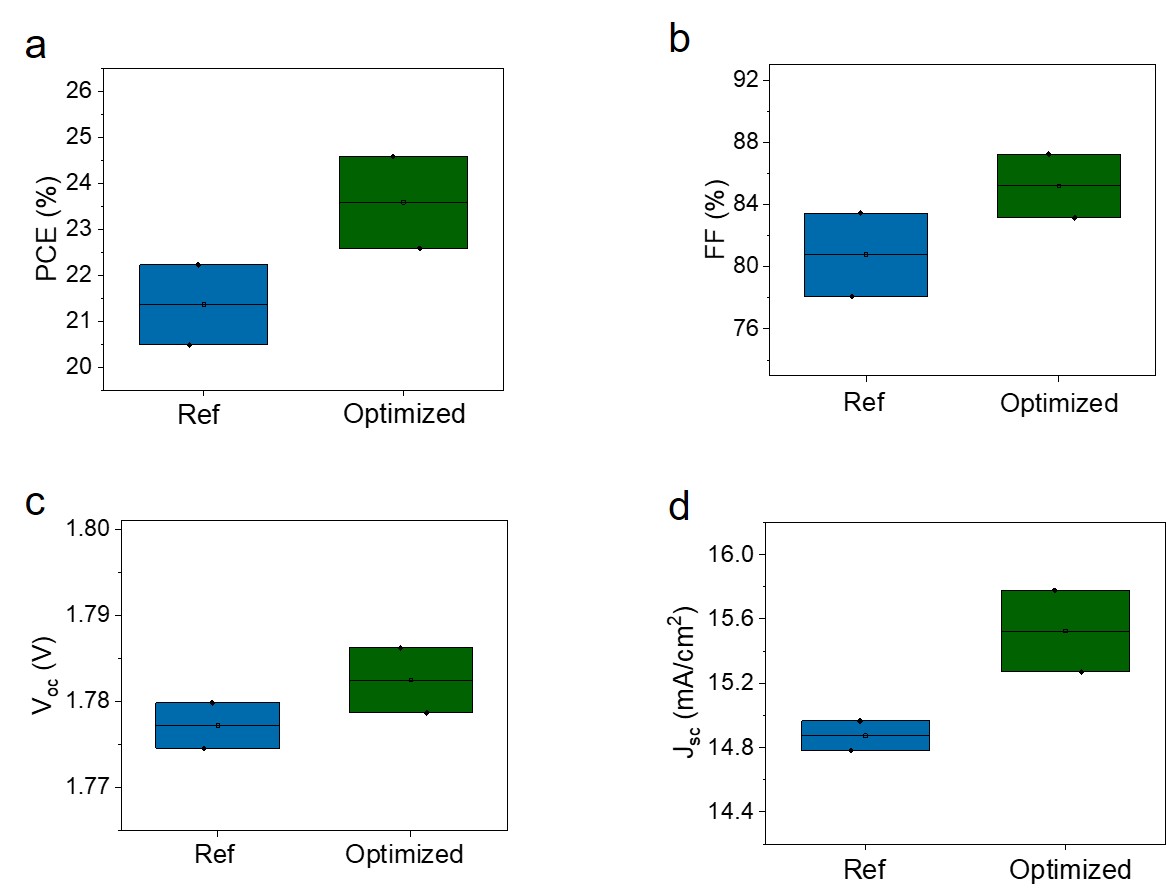


Supplementary Fig. 1│ Box comparison of performance of two samples before and after optimization. shrinking parasitic absorption losses and passivating the C60/perovskite interface.


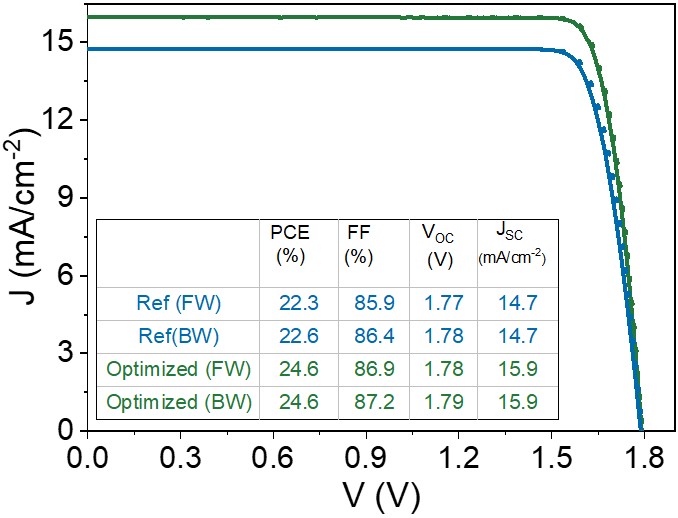


Supplementary Fig. 2│ comparison of J-V results of samples before and after optimization. including the performance parameters of samples.

Supplementary Fig. 3│ MPP Tracking of 2T tandem solar cell with LiF passivation (black) and PDAI_2_ passivation (red) The solar cells were tracked in nitrogen at 25°C for 150 hours.


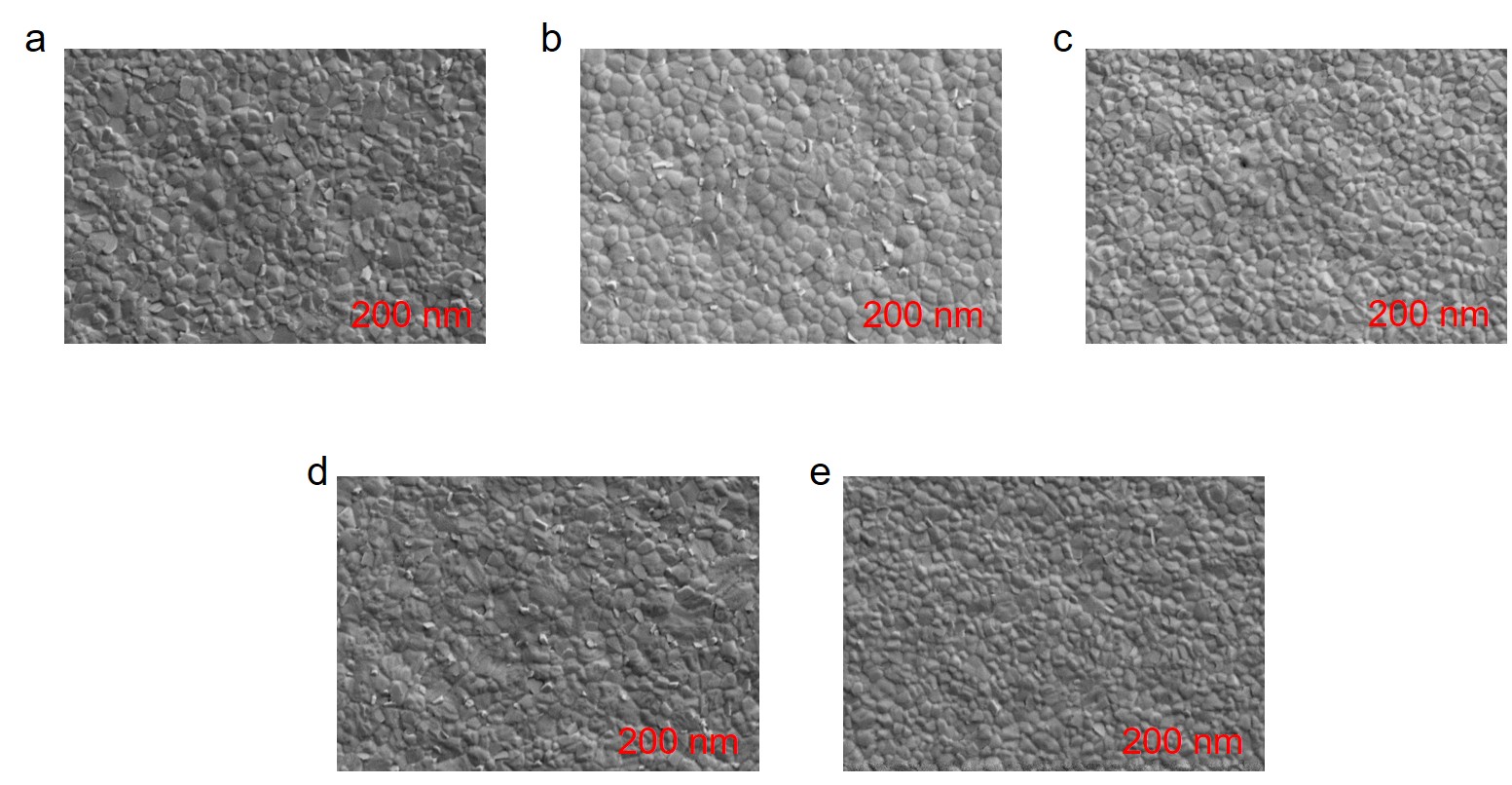


Supplementary Fig. 4│ Top-view SEM images of perovskite films. Images (a)–(e) represent samples with increasing bandgaps of 1.52, 1.58, 1.63, 1.68, 1.73 eV, respectively.

Supplementary Fig. 5│ XRD patterns of the perovskite films.

Supplementary Fig. 6│ FF changes of subcells, as well as 2T and 3T tandem cells, with five different perovskite bandgaps. The lighter and darker color expresses the lower and higher respective performance parameter of a cell, respectively.

Supplementary Fig. 7│ EQE spectra of the perovskite (black) and silicon (blue) subcells for different bandgaps. Including the integrated J_SC_ of the corresponding subcells. There were three devices for the bandgaps of 1.52, 1.58, and 1.63 eV, and one for the bandgaps of 1.68 and 1.73 eV.

# Supplementary Fig. 8│ Dependence of 2T and 3T performances on perovskite bandgaps.


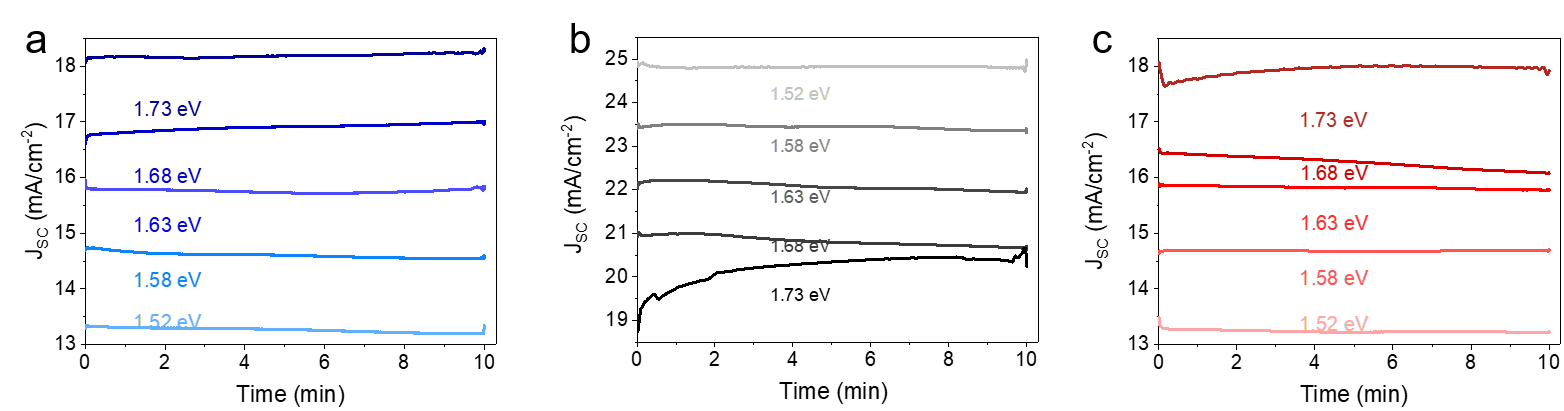


**Supplementary Fig. 9**│10-minute MPP track of current density of all bandgaps. The subcells and 2T tandem cells were measured non-encapsulated in air. Temperature and humidity were not controlled, 20-30 °C and 25-35%, respectively.


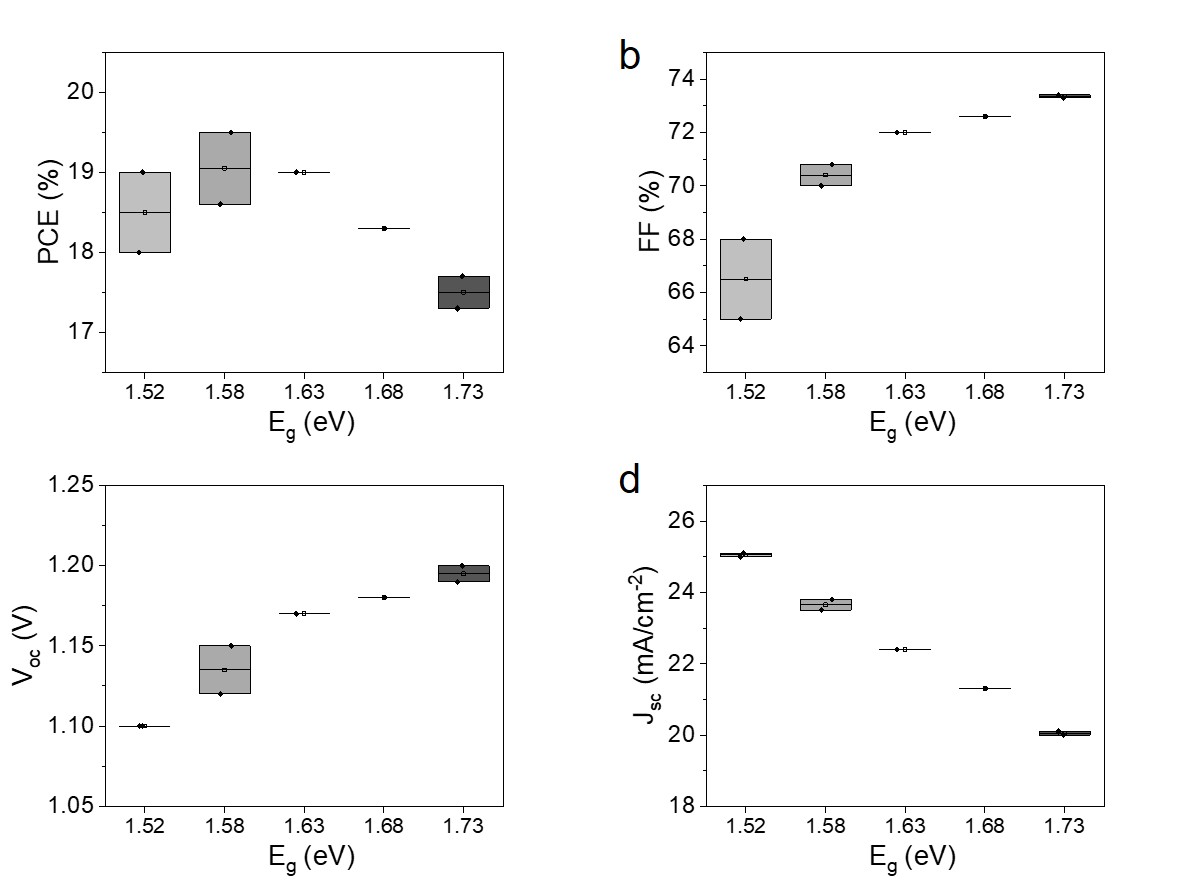


Supplementary Fig. 10│ Box comparison of one or two of perovskite subcell performance with different bandgaps. PCE, FF, V_OC_, and J_SC_, respectively. The respective T and Z contacts are used to measure 2T mode of tandem solar cells


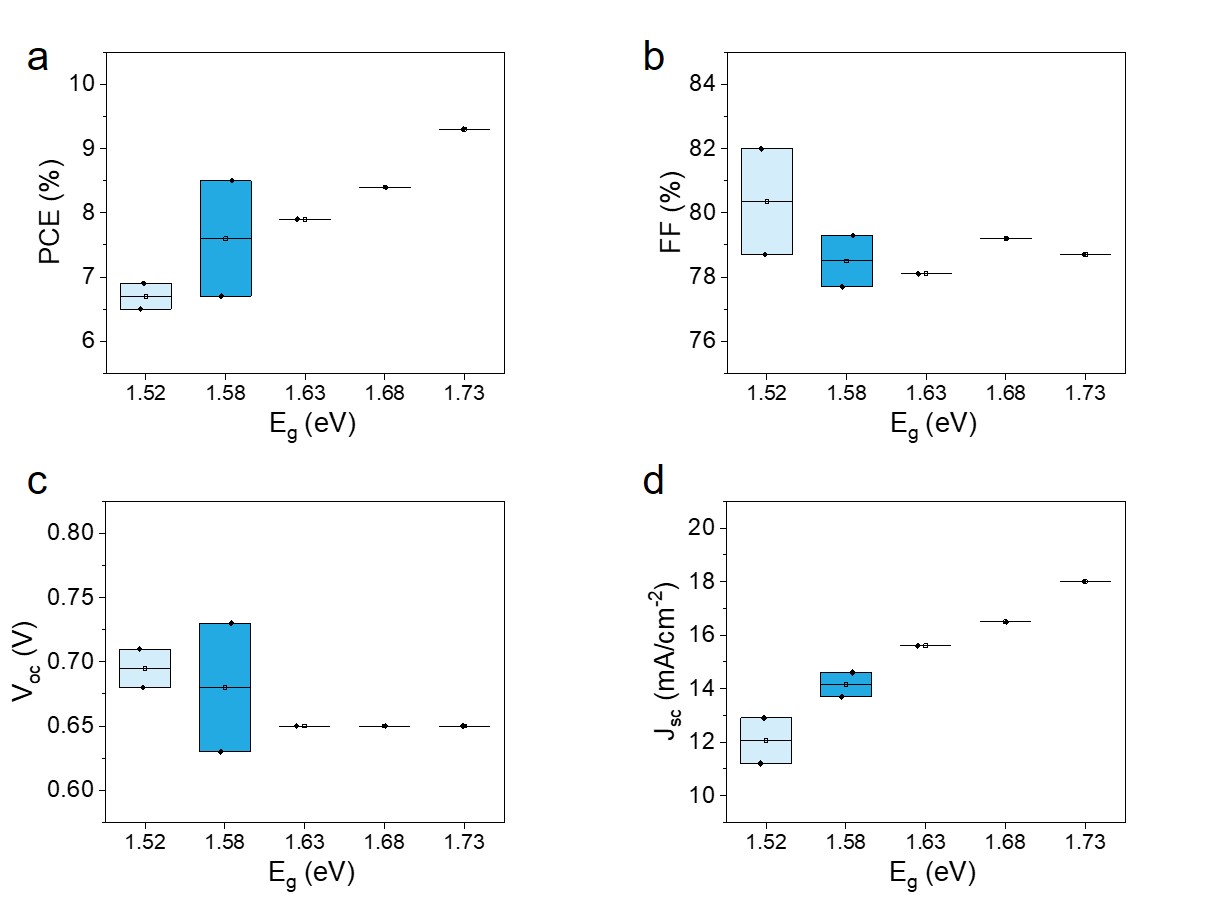


Supplementary Fig. 11│ Box comparison of one or two of silicon subcell performance for different bandgaps. PCE, FF, V_OC_, and J_SC_, respectively. The respective R and Z contacts are used to measure 2T mode of tandem solar cells


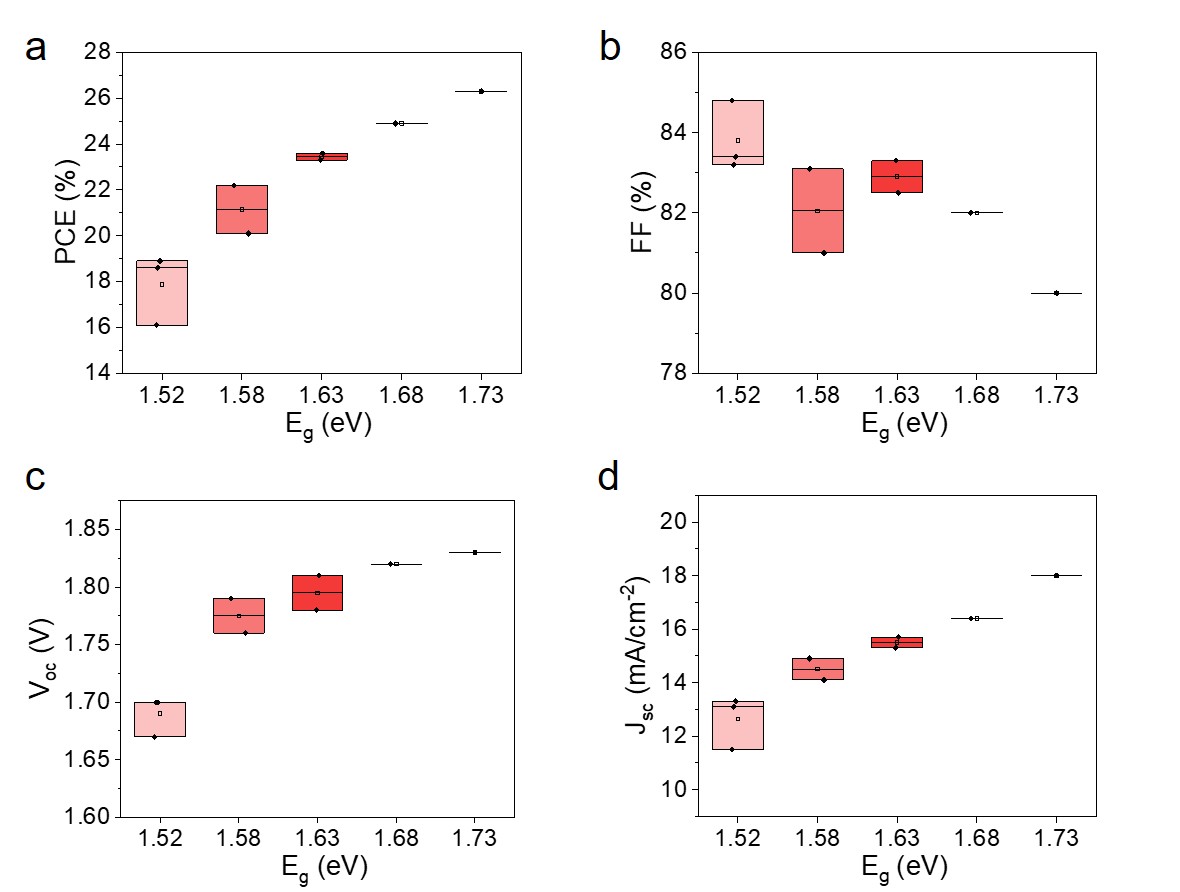


Supplementary Fig. 12│ Box comparison of one or two of 2T tandem device performance for different bandgaps. PCE, FF, V_OC_, and J_SC_, respectively. The respective R and T contacts are used to measure 2T mode of tandem solar cells


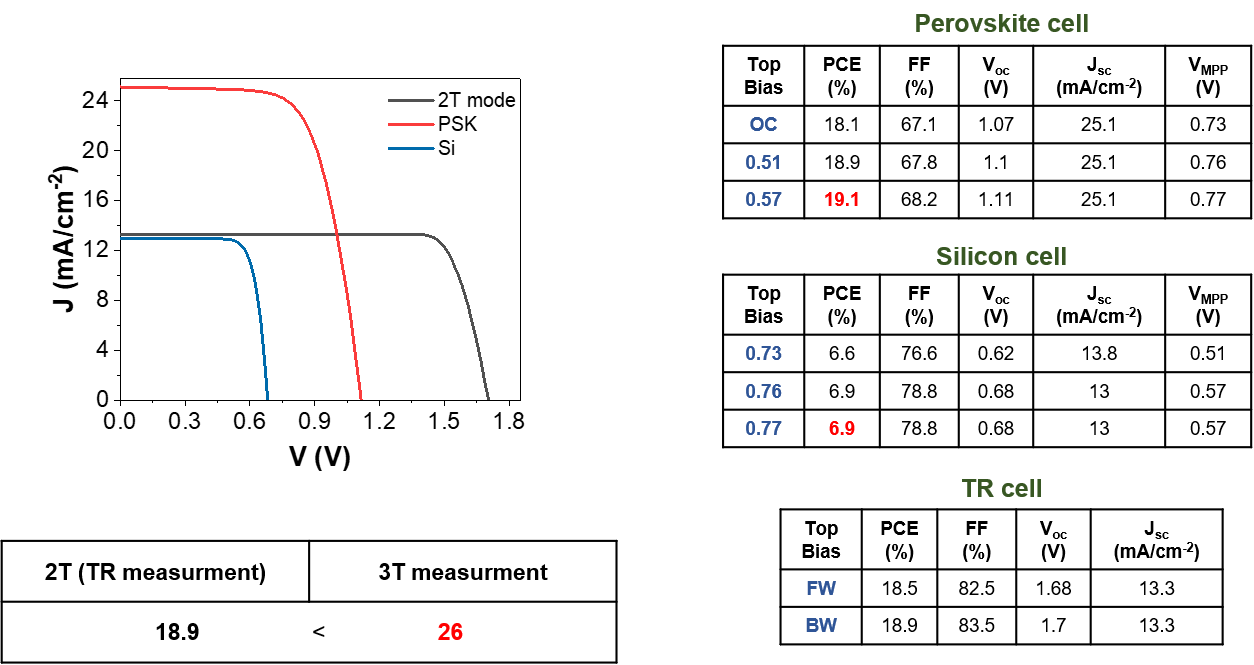


Supplementary Fig. 13│ subcells, 2T, 3T devices performance data of 1.52 eV bandgap, along with their corresponding J-V data. Tables represent V_MPP_ changes and performance parameters in an iterative measurement.


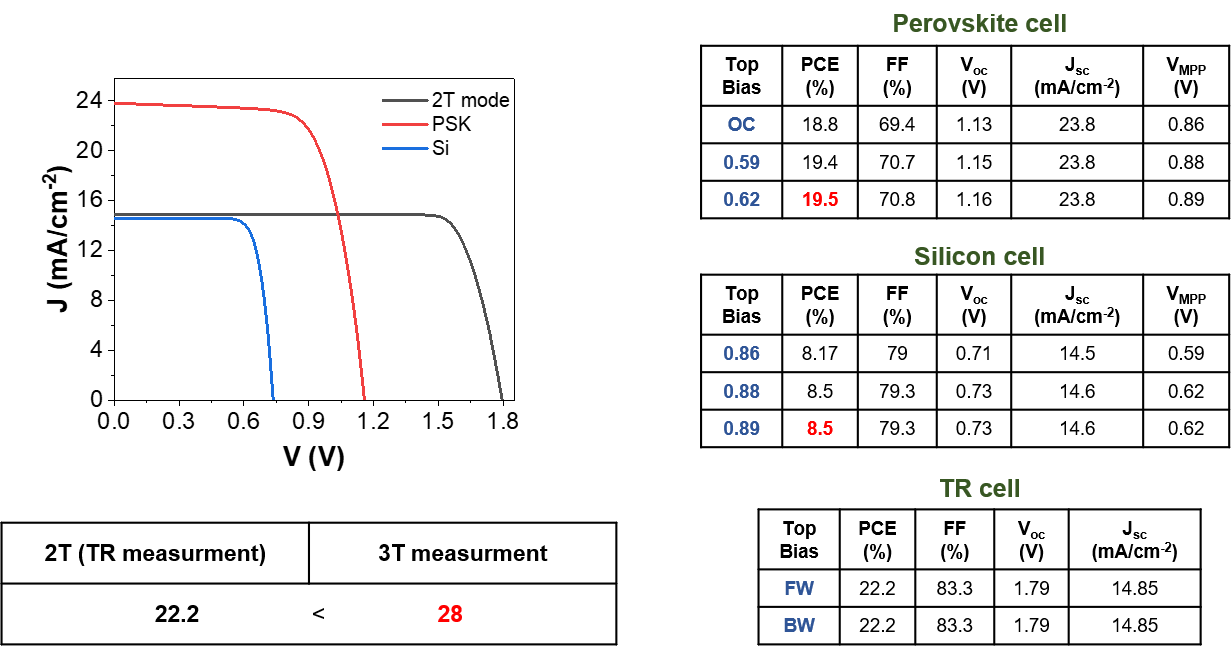


Supplementary Fig. 14│ subcells, 2T, 3T devices performance data of 1.58 eV bandgap, along with their corresponding J-V data. Tables represent V_MPP_ changes and performance parameters in an iterative measurement.


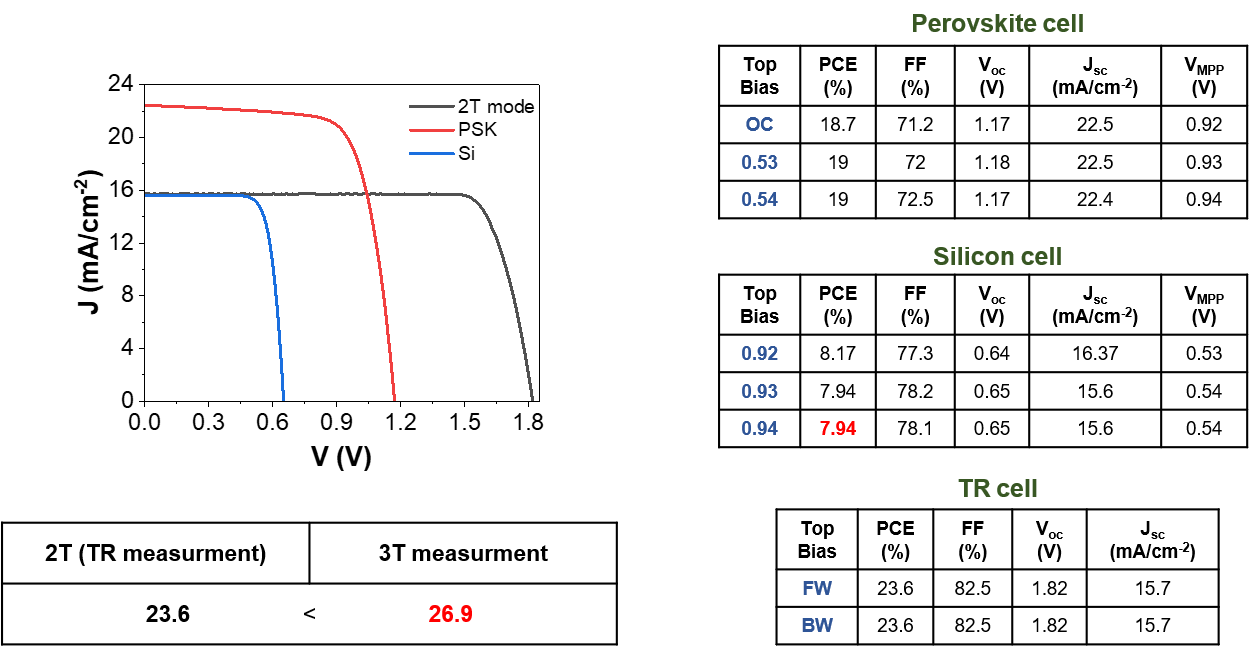


Supplementary Fig. 15│ subcells, 2T, 3T devices performance data of 1.63 eV bandgap, along with their corresponding J-V data. Tables represent V_MPP_ changes and performance parameters in an iterative measurement.


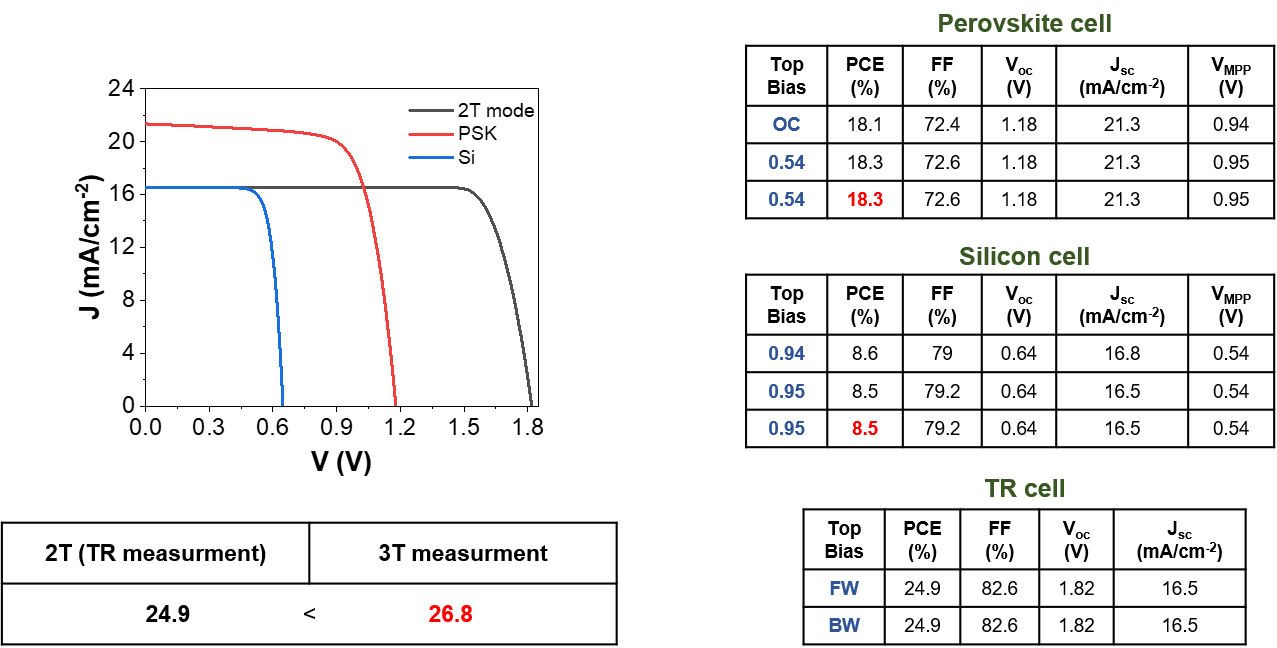


Supplementary Fig. 16│ subcells, 2T, 3T devices performance data of 1.68 eV bandgap, along with their corresponding J-V data. Tables represent V_MPP_ changes and performance parameters in an iterative measurement.


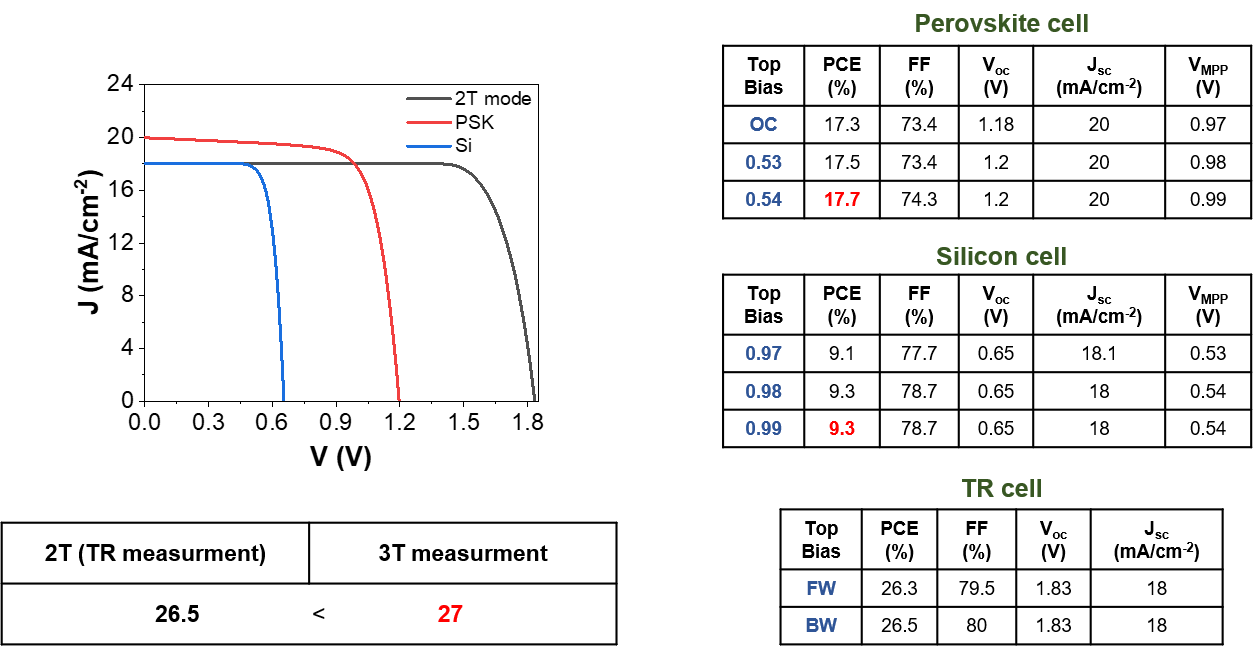


# **Supplementary Fig. 17│ subcells, 2T, 3T devices performance data of 1.73 eV bandgap, along with their corresponding J-V data.** Tables represent V_MPP_ changes and performance parameters in an iterative measurement.

# Supplementary Fig. 18│ solar simulator spectra

Supplementary Fig. 19│ Measured EQE spectra (dotted line) and simulated EQE spectra (solid line) of subcells. The perovskite and silicon subcells are indicated by black and blue colors, respectively. **T**he bandgaps of perovskite is 1.73 eV.

|  | **Parameters** | **Simulated** | **Measured** |
| --- | --- | --- | --- |
| **Perovskite** | J_SC_ (mA/cm^-2^) | 20 | 19.66 |
|  | V_OC_ (V) | 1.2 | 1.18 |
|  | FF (%) | 74.3 | 79 |
|  | PCE (%) | 17.7 | 18.3 |
| **Silicon** |  |  |  |
|  | J_SC_ (mA/cm^-2^) | 18 | 18.3 |
|  | V_OC_ (V) | 0.65 | 0.65 |
|  | FF (%) | 78.7 | 81 |
| **2T** | PCE (%) | 9.3 | 9.6 |
|  | J_SC_ (mA/cm^-2^) | 18 | 18.3 |
|  | V_OC_ (V) | 1.83 | 1.83 |
|  | FF (%) | 79.5 | 79.2 |
|  | PCE (%) | 26.3 | 26.5 |
| **3T** | PCE (%) | 27 | 27.9 |

Supplementary Table. 1│ Performance parameters of Measured and simulated EQE.


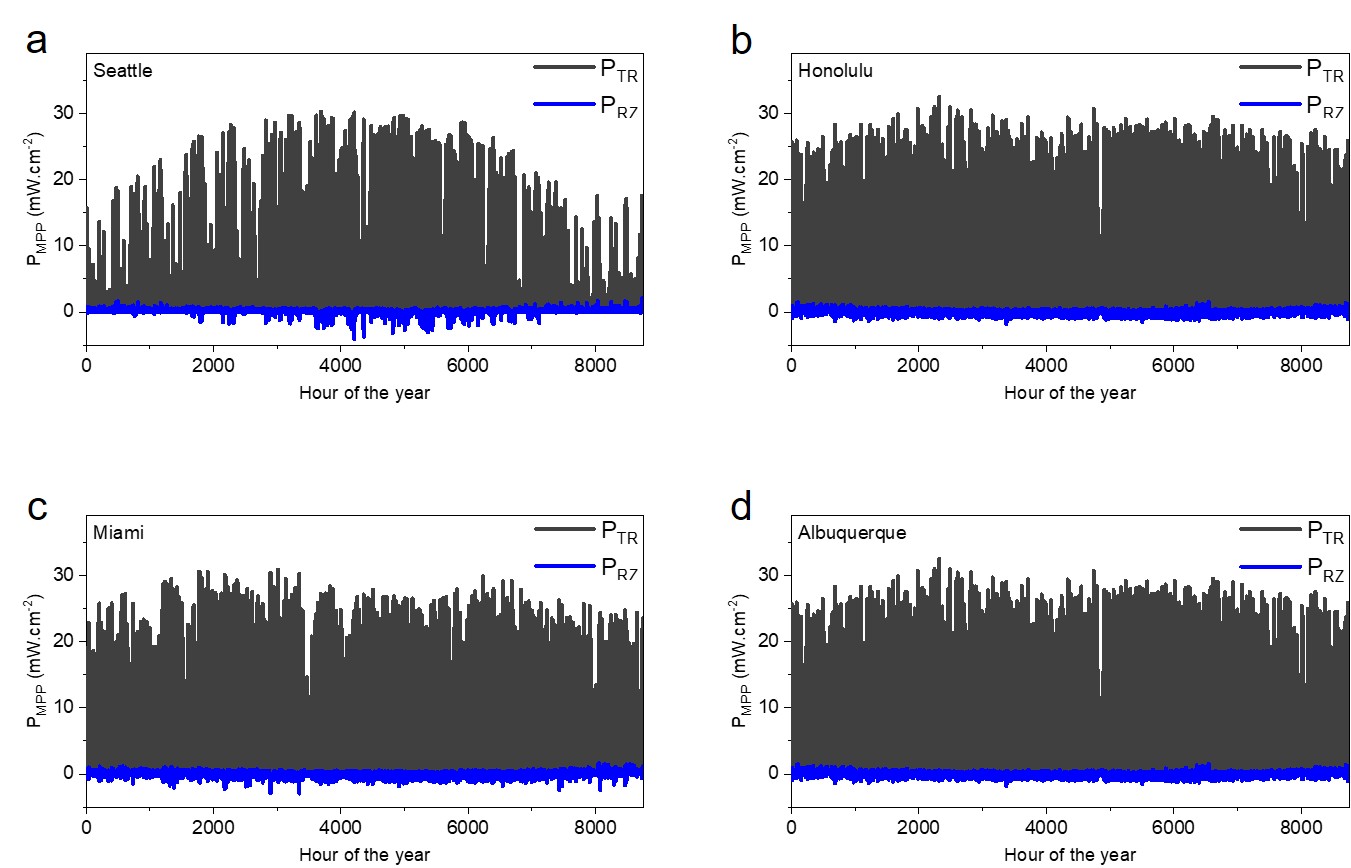


# Supplementary Fig. 20│ Power generation proﬁle in the RZ and RT circuits of the 3T solar cell in Seattle, Honolulu, Miami, and Albuquerque for a perovskite layer with a band gap of 1.73 eV.
